# Application of extracorporeal shock wave therapy in nervous system diseases: A review

**DOI:** 10.3389/fneur.2022.963849

**Published:** 2022-08-17

**Authors:** Juan Guo, Hong Hai, Yuewen Ma

**Affiliations:** Department of Rehabilitation Medicine, The First Affiliated Hospital of China Medical University, Shenyang, China

**Keywords:** extracorporeal shock wave therapy, peripheral nervous system diseases, central nervous system diseases, mechanotransduction, cost-effectiveness, neural tissue regeneration

## Abstract

Neurological disorders are one of the leading causes of morbidity and mortality worldwide, and their therapeutic options remain limited. Recent animal and clinical studies have shown the potential of extracorporeal shock wave therapy (ESWT) as an innovative, safe, and cost-effective option to treat neurological disorders. Moreover, the cellular and molecular mechanism of ESWT has been proposed to better understand the regeneration and repairment of neurological disorders by ESWT. In this review, we discuss the principles of ESWT, the animal and clinical studies involving the use of ESWT to treat central and peripheral nervous system diseases, and the proposed cellular and molecular mechanism of ESWT. We also discuss the challenges encountered when applying ESWT to the human brain and spinal cord and the new potential applications of ESWT in treating neurological disorders.

## Introduction

ESWT originates from high-intensity extracorporeal shock wave lithotripsy (ESWL), a well-established treatment method for urolithiasis (1, 2). It was found later that a decrease in the energy of ESWL could produce more beneficial effects on human tissues. As a new disease treatment option, ESWT offers many benefits. First, because ESWT has a lower energy intensity than that of traditional ESWL (3, 4), it is generally considered safer and non-invasive and has fewer side effects such as pain, small and superficial ecchymosis, and mild numbness (5). Second, ESWT is simple and relatively easy to operate. For example, when ESWT is used, physicians just need to first determine the area targeted by ESWT, which can be achieved by examining the lesion location. Then the gel is placed on the skin of the to-be-treated area. Thereafter, physicians can simply select and adjust the treatment according to patients' pain, tolerance, and other factors to achieve the best result. Clearly, the ESWT procedure is much simpler than surgery. Third, in terms of convenience and cost-effectiveness, ESWT has a long treatment interval with short treatment times. Thus, patients do not need to spend much time or money on ESWT. Because of these beneficial characteristics, ESWT has been a therapeutic option for the treatment of many musculoskeletal diseases (6, 7), including plantar fasciitis (8–10), calcific shoulder tendinopathy (11), tennis elbow (12), trigger finger (13), knee osteoarthritis (14), and bone non-union (15–17). ESWT has also been utilized to treat wounds (18–21), urological diseases (22–24), and male erectile rejuvenation (25).

Neurological disorders consist primarily of central nervous system diseases and peripheral nerve injury. Central nervous system diseases can be a significant burden to the family and society because of their severe conditions (26), while peripheral nerve injury is difficult to cure and often leads to pain and functional disorders. Drug therapy and surgery are standard options to treat these conditions, but drug therapy often produces limited clinical benefits, while surgery may necessitate supplementary therapy to achieve better rehabilitation. Clearly, treatment of these diseases remains a challenge and there is a need for new options for their treatment. Recently, considerable animal and clinical research have been carried out to explore the use of ESWT to treat neurological disorders and the results are very encouraging because they demonstrated that ESWT has the potential to become a valuable therapy for neurological disorders. Despite the fact that there is no general medical guide currently available, which can guide physicians in using ESWT to treat neurological disorders, physicians at many hospitals have already utilized ESWT clinically to treat patients with neurological disorders such as limb spasm and peripheral nerve diseases.

To better inform the physicians about the potential of ESWT as a therapeutic option for the treatment of neurological disorders, in this review, we describe the properties of ESWT and the cellular and molecular mechanism proposed to elucidate how the neural tissues respond to ESWT, review the animal and clinical studies involving ESWT, and discuss the challenges encountered and new prospects for the use of ESWT as a therapeutic option for treatment of neurological disorders.

## Principles of ESWT

**Figure 1:**
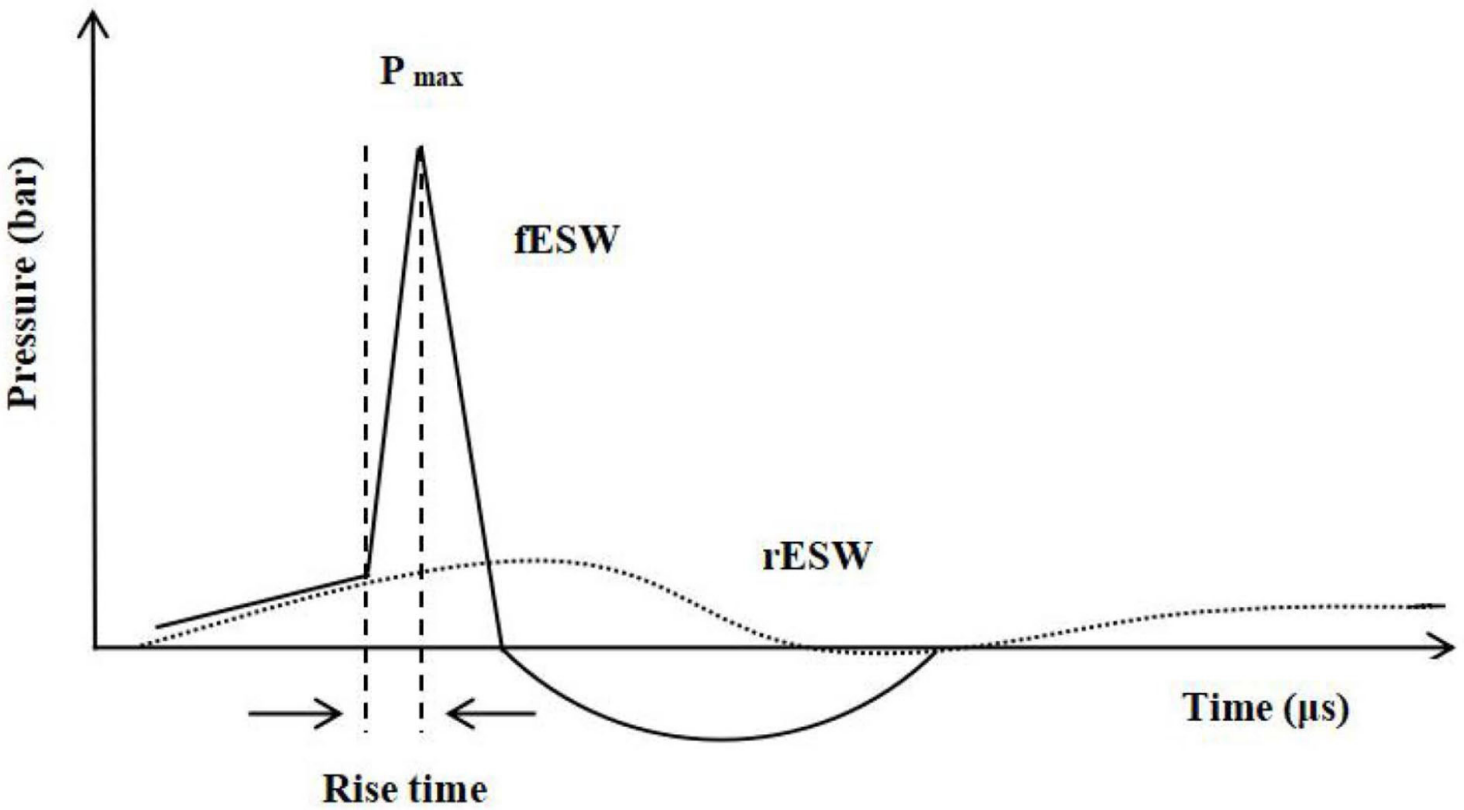
—Waveform characteristics of shock wave (fESW): a short rise time, high peak pressure, and non-linearity. Pmax, Pressure maximum. ...Waveform characteristics of pressure wave (rESW).

ESWT is a form of mechanotherapy with a peak pressure of about 1,000 times more than ultrasound therapy (27). ESWT can be classified into focused ESWT (fESWT) and radial ESWT (rESWT) based on the wave patterns used (28). In general, a focused extracorporeal shock wave (fESW) is generated electrohydraulically, electromagnetically, or piezoelectrically, followed by converging it into a focal tissue zone (29). As an acoustic wave, fESW is characterized by its high pressure of more than 1,000 bar (100 MPa), an extremely short rise time (<10 ns), a short duration (<10 ms), and a broad frequency spectrum (16–20 MHz) (30–32). Unlike fESW, radial extracorporeal shock wave (rESW) does not possess the shock wave characteristics of a short rise time, a high peak pressure, and non-linearity. Some scholars even call “rESW” “radial pressure waves” because rESW uses the energy generated from compressed gas to drive the bullet body to the treated tissue area in a pulsed manner (29, 33, 34) (Figure 1).

The major parameters that affect the treatment effectiveness of ESWT include air pressure (unit: bar), energy flux density (EFD) (unit: mJ/mm^2^), number of pulses (unit: pulses), and frequency (unit: Hz) (35, 36). EFD is the parameter that indicates the energy intensity of shock waves in a unit area. It was suggested that ESWT should be classified into low (<0.08 mJ/mm^2^), medium (<0.28 mJ/mm^2^), and high (<0.60 mJ/mm^2^) energy according to the energy (intensity) value of its EFD (37).

The effectiveness of ESWT also depends on the penetration depth of ESWT. The probe of the handpiece used in an ESWT device dictates the penetration depth of ESWT because each probe has a specific penetration depth range. It is noted that the ESWT device makers provide various handpieces of ESWT, sometimes making it difficult for physicians to select which handpieces to be used clinically. Compared with rESWT, fESWT produces a shock wave with a higher EFD and its probe can penetrate deeper treatment areas (38, 39). To illustrate, rESWT is generally safer when used to treat superficial bone fracture non-union like tibia (15). In contrast, when deeper lesions such as femoral head necrosis are treated, fESWT is frequently used because its higher EFD could lead to a better therapeutic result (40). In general, fESWT can reach tissues that are as deep as 12 cm, while the penetration depth of rESWT is only 3–4 cm deep (41).

It is generally believed that ESWT produces two significant physical effects on tissues. The first physical effect is called “mechanotransduction”, which refers to the capability of ESWT to produce the shear and pressure forces (42). Mechanotransduction can reduce and even reverse injury to damaged tissues, and mechanically promote homeostasis in healthy tissues at the molecular, cellular, and tissue levels. Mechanotransduction is generally considered a direct physical effect because it directly acts on tissues (43). It is suggested that ESWT-induced direct mechanical perturbations might be transmitted to tissues, affecting cell membrane polarization, radical formation, cell proliferation, and growth factor production (7, 44). It is also proposed that cells could sense mechanical forces and transmit mechanical stimuli into biochemical signals, which then lead to modulation of the functions of cells in turns, such as migration, proliferation, and differentiation, and even maintaining cytoskeletal structure and homeostasis, in which mechanotransduction plays an essential role (45). It has been suggested that the action of ESWT on the mechanotransduction signaling pathway consists of four major phases (46): the first phase is the mechanocoupling phase, which converts external mechanical signals (shock waves) to mechanical signals near the cell; the second phase is called biochemical coupling, meaning that mechanical signals are transduced into biochemical signals, thus leading to changes in the gene and/or protein level; the third phase is the signal transmission, which sends biochemical signals from sensor cells to the effector cells; the last phase is the responses of the effector cells. It is the cytoskeleton that mainly senses the mechanical stimuli of shock waves during the first phase (47), while certain cell membrane proteins are activated in the second phase, causing a downstream signaling response. For example, it has been reported that the proteins like Piezo serve as mechanical sensitive ion channels (48). Under the impact of ESWT, these mechanical sensitive ion channels become activated to transfer biochemical signals into cells, causing changes in downstream “mechanotransduction” proteins. It is noted that the exact mechanism of mechanotransduction is still not well understood when ESWT is used to treat neurological disorders. More studies are warranted to better understand the treatment mechanism of mechanotransduction for this direct physical effect, which could potentially lead to new approaches to the treatment of neurological disorders by ESWT (43).

The second physical effect is called cavitation, which refers to the rapid formation, expansion, and subsequent implosion of air bubbles due to the negative pressure (49). Shock waves could produce high stress at the boundary interface between the body tissues and generate the tensile forces that lead to cavitation, which is regarded as an indirect mechanical force of shock waves (50). The process in which the growth and collapse of acoustic waves including shock waves induce bubbles in liquid is called acoustic cavitation. Acoustic cavitation not only breaks calcific deposits, but also excites nerves, stimulates axons, and reduces pain (51). There are many small cavitation bubbles in the liquid between the body tissues. When the high-energy shock wave acts on the tissues, it can cause the rapid decline of the bubble surface tension and lead to the elastic deformation between the tissues, thus relaxing the local soft tissue adhesion and reducing the local adhesion symptoms. Although the cavitation effect is likely to damage some normal cells, the cavitation is predictable and regulatable, and thus it can be used for the treatment of certain diseases. For example, in the nervous system, a shock wave with high energy may cause traumatic encephalopathy, but the cavitation effect induced by the appropriate dose of the shock wave can make the drug effectively pass through the blood-brain barrier (52), which is conducive to tumor blood vessel blockage. In addition, when it comes to the ability of the cavitation induced by ESWT to excite nerves, researchers found that cavitation could excite neural tissues through the interaction between shock waves and bubbles to generate the action potential (53).

In summary, both mechanotransduction and cavitation may play a major role in introducing shock waves to tissues and triggering physiological actions at the molecular and tissue levels, which then produce beneficial biological events such as tissue regeneration and repairment, angiogenesis, pain relief, metabolic activation, and anti-inflammation, leading to beneficial therapeutic results as demonstrated in many animal and clinical studies.

## Animal studies of ESWT

### Sciatic nerve injury

**Figure 2:**
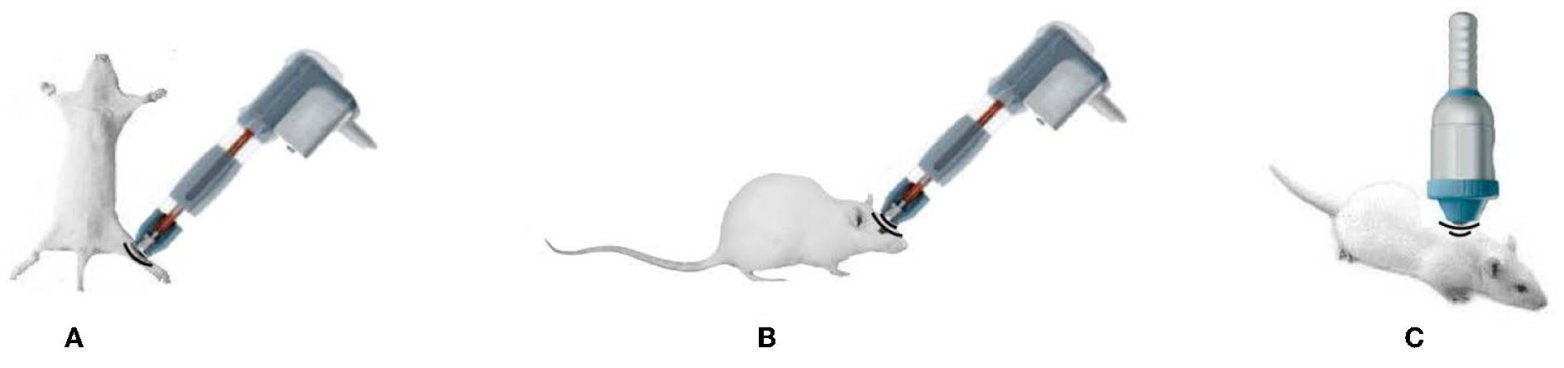
Animal studies of the use of ESWT in sciatic nerve **(A)**, brain **(B)**, and spinal cord **(C)**.

Sciatic nerve injury is a peripheral nerve disorder. When applied to the animals with sciatic nerve injury, ESWT was found to be able to lead to the recovery of peripheral nerve injury (54–56) (Figure 2A). For example, in an animal study, medium-energy ESWT (a frequency of 3 Hz and energy flux density of 0.09 mJ/mm^2^, 300 pulses) was employed to treat rats' sciatic nerve damage. This study showed that the rats in the ESWT group had a significantly increased sciatic functional index (SFI) (57). Animal studies also suggested that it is essential to use the appropriate dose of ESWT to treat sciatic nerve injury. Fu et al. (56) established a rat model of chronic nerve compression of sciatic injury by applying ESWT of 1.0, 1.5, 2.0, and 2.5 bar pressure to rats. Their results showed that ESWT with 1.0 bar pressure led to the best improvement in relieving pain in rats, suggesting that there may be an energy saturation effect on the therapeutic effectiveness when ESWT is used to treat nerve compression injury. Another study showed that the pulses of 300 shock waves led to the best therapeutic result, enhancing nerve function recovery when ESWT was used to treat injured sciatic nerve (58).

### Central nervous system diseases

Because of the concerns about the potential damage to the human brain or spinal cord by ESWT, few clinical studies were carried out to explore the clinical use of ESWT in the treatment of human central nervous system diseases. However, animal studies have shown that ESWT was effective when treating central nerve damage. In general, cerebral infarction and spinal cord injury (SCI) were the focus of the animal studies (Figures 2B,C). In one study, ESWT was used to treat stroked rats' brains. The result showed that ESWT could inhibit neurological dysfunction of rats after acute ischemic stroke and was able to reduce brain infarct volume (59). Kang et al. (60) also used ESWT (2.0 bar, 200 pulses, 10 Hz) to treat rats' brains with cerebral infarction. Their result showed that appropriate doses of ESWT could improve cerebral blood flow effectively, increasing the neurological function of rats without side effects. In a study of treating chronic SCI by ESWT, Lee et al. (61) found that rats' behavioral tests were improved when the stem cell therapy was combined with ESWT. In addition, fESWT at three energy levels (level 1, 0.01 mJ/mm^2^; level 2, 0.04 mJ/mm^2^; and level 3, 0.11 mJ/mm^2^) was applied at 1,000 pulses to rats' spinal cords. Histological examination has shown that there were no neurological impairments when fESWT was applied at these energy levels. It was also reported that when ESWT was used to treat spinal cord injury rats, it reduced the neural tissue damage, enhanced the effectiveness of neuroprotection, and improved motor function without any detrimental effect (62–64).

## Clinical studies of ESWT

### Carpal tunnel syndrome

Carpal tunnel syndrome (CTS) is an entrapment neuropathy of the median nerve characterized by pain and paresthesia in median nerve innervated areas (65). There are many conservative interventions such as therapeutic ultrasound, wrist splinting, and steroid injections to treat CTS (66), which reduce the intracarpal tunnel pressure by reducing inflammation reactions. However, these treatment approaches generally produce short-term therapeutic efficacy (67). Recently, ESWT was shown to be a promising therapeutic method for the treatment of peripheral nerve injury because of its therapeutic efficacy. Ke et al. (68) used ESWT to treat the area of the median nerve walk from the pea bone level to the proximal end of the carpal tunnel entrance with 2,000 pulses and 4 bar pressure at a frequency of 5 Hz once per week. They found that ESWT was a promising therapeutic strategy for patients with mild to moderate CTS and multiple-session ESWT produced cumulative clinical results. Other studies have shown that ESWT improved pain symptoms in patients with CTS (69) and reduced the sensory latency of the median nerve (70). ESWT can also potentially be an alternative conservative strategy. Compared with other conservative strategies, ESWT demonstrated good short-term treatment efficacy and produced long-term therapeutic effects on CTS. It was reported that the optimal therapeutic result can be achieved if the probe of ESWT is placed perpendicularly on the median nerve and the EFD level is low or medium when CTD is treated by ESWT. In general, both fESWT and rESWT can provide safe and good outcomes when used to treat CTS (71–75) (Table 1).

**Table 1:** Summary of the applications of ESWT in CTS.

**Treatment**	**Group mode**	**Study design**	**Reference**	**Intervention and** **ESWT Protocol**	**Follow-up**	**Outcome**
rESWT	wrist splint group (*n =* 47), splint+rESWT group (*n =* 47), rESWT group (*n =* 45), splint+ placebo rESWT group (*n =* 50)	RCT	71	– ESWT (1,000 shocks, 0.05 mJ/mm^2^ at 5 Hz; 3 sessions, 1 week between sessions) – wrist splint	3 months	Pain and functionality significantly improved in ESWT groups; A more remarkable improvement in the hand function and electrophysiological measures was observed in the group with ESWT and a combined wrist splint.
ESWT	ESWT group (*n =* 30), Local corticosteroid injection (LCI) group (*n =* 25)	RCT	72	– ESWT (1,000 shocks, 1.5 bar at 6 Hz; 3 sessions, 1 week between sessions) – LCI	3 weeks 9 weeks 12 weeks	Pain, function and median nerve sensory nerve action potential distal latency improved in the 9 and 12-week follow-ups for the ESWT group.
rESWT	single dose rESWT group (n = 13), LCI group (n = 12)	RCT	73	– ESWT (5,000 shocks, 4 bar at 15 Hz; 1 session) – LCI	1 week 4 weeks 12 weeks 24 weeks	Significantly greater improvement in symptom severity, functionality and pain at weeks 12 to 24 in the rESWT group.
fESWT	60 patients with CTS were examined in ESWT group and control group	RCT	74	– ESWT (800–1,100 shocks, 0.05–0.15 energy; 4 sessions, 1 week between sessions) – Conservative treatment including wrist splint; nonsteroidal anti-inflammatory drugs; Vitamin B1	3 months 6 months.	Pain and function significantly improved in the ESWT group after 3 months and 6 months of treatment.
fESWT	shock wave group (*n =* 34), nutraceutical group (*n =* 26)	RCT	75	– ESWT (1,600 shocks, 0.03 mJ/mm^2^ at 4 Hz; 3 sessions, 1 week between sessions) -Nutraceuticals composed of ALA, GLA, and Echinacea	6 months	ESWT provided an improvement comparable to nutraceuticals on pain and functional ability in patients with CTS.

ESWT, extracorporeal shock wave therapy; fESWT, focused extracorporeal shock wave therapy; rESWT, radial extracorporeal shock wave therapy; CTS, Carpal Tunnel Syndrome; RCT, randomized clinical trial; LCI, Local corticosteroid injection; ALA, α-Linolenic acid; GLA, γ-linolenic acid.

### Postherpetic neuralgia

Postherpetic neuralgia (PHN) is a common type of neuralgia related to neuronal damage and can seriously affect the quality of life due to the pain and poor response to the currently available treatments (76, 77). The main approaches to PHN management are medication and invasive interventional therapies. However, these methods have many adverse effects (76). Physicians have used non-invasive ESWT to treat the pain area of PHN. It was found that both fESWT and rESWT could reduce pain (78) and produced effective outcomes with few side effects when PHN was treated by ESWT. For example, a clinical study has shown that when ESWT [rESWT: R15 probe (radius of 15 mm), 1–4 bar, 5,000–7,000 pulses, 10 Hz] was used to treat patients with PNH, ESWT reduced pain and improved life quality compared with conventional therapies (76). Another clinical study showed that the medium-energy ESWT with 6 sessions (fESWT, EFD of 0.09–0.16 mJ/mm^2^, frequency of 5 Hz, and 2,000 pulses) could reduce the numerical rating scale of PNH, suggesting that fESWT could alleviate the skin pain and itch (77).

### Trigeminal neuralgia

Trigeminal neuralgia (TN) is a severe type of neuralgia that occurs in the distribution area of the facial trigeminal nerve (79). Persons with TN tend to experience a series of painful symptoms. Zhang et al. (80) reported that a woman with primary TN was treated by ESWT two times a week for 3 years with 3,000–6,000 pulses at a frequency of 10 Hz with an air pressure of 1.4–1.5 bar and the treatment sites were centered on the anterior ear region (the projection area of the trigeminal ganglion surface) and the pain area of the upper and lower jaw. The patient's pain was relieved after the 8-week treatment and the patient's life quality was significantly improved after the 3-month treatment. This study showed that ESWT produced good treatment efficacy, demonstrating the potential and feasibility of applying ESWT to treat diseases involving human facial and peripheral nerves.

### Central nervous system diseases

Current clinical studies of ESWT mainly focus on peripheral nervous system diseases and much fewer studies have been carried out to explore the use of ESWT to treat human central nervous system diseases because of the concern about potential injury of the brain and spinal cord by ESWT. However, one study has already shown that ESWT could be applied to the brain of patients with unresponsive wakefulness syndrome to improve consciousness (81). In that study, it was found that the skull absorbs most of the shock wave energy and only about 10% of the total energy actually reached the brain. It was reported that ESWT could be effective if the actual energy that reached brain tissues was in the range of 0.01–0.02 mJ/mm^2^ (81). Werner et al. (82) also used fESWT to treat brain stem (0.1 mJ/mm^2^, 4,000 pulses, 6 Hz) three times a week over four weeks with a penetration depth of the focus of approximately 5.5 cm. They reported that ESWT could stimulate vigilance in patients with unresponsive wakefulness syndrome and suggested that the use of ESWT with a relatively low EFD may cause fewer side effects when ESWT is used to treat human brain diseases (81). Moreover, the animal studies discussed above have also demonstrated the feasibility as well as safety of ESWT when it was employed to treat central nervous system diseases. Nevertheless, when ESWT is applied to the human brain or spinal cord, the energy dose of ESWT is still a critical issue for physicians to consider and more studies are needed to examine the potential adverse effects of ESWT on human brain tissues and determine the optimal ESWT energy level to be used so that ESWT can be used effectively as well as safely to treat the diseases involving human brain tissues.

Limb spasticity is one of the common neurological symptoms of central nervous system diseases, which include stroke, spinal cord injury, cerebral palsy (CP), and multiple sclerosis (MS) (83). The existence of spasticity may be associated with hyperexcitation of distraction reflex due to motor neuron lesion (84). Studies showed that ESWT improved upper and lower spasticity in post-stroke patients (85, 86). Wu et al. (87) compared the therapeutic effect of rESWT with that of fESWT on post-stroke spastic equinus and found that both increased the passive motion of the ankle joint and the contact area of the foot. ESWT was also safe and effective when used to treat limb muscle spasms in children with cerebral palsy (88–90). Because rESWT is less painful, cheaper, and more comfortable when used to treat spasticity in comparison with fESWT and rESWT can reach spastic muscles (88), most clinical studies use rESWT to treat spasticity. Leister et al. (91) suggested that ESWT could be used to treat acute traumatic spinal cord injury and they have developed a protocol for using ESWT to treat motor and sensory impairment as well as spasticity caused by spinal cord injury. Since ESWT is non-invasive, it has been reported that ESWT could also be a good option to treat spasticity in people with multiple sclerosis (92). In short, ESWT, especially rESWT, has the potential to reduce spasticity in various central nervous system diseases. Table 2 summarizes various applications of rESWT (88, 90, 93–96).

**Table 2:** The application of rESWT to treat spasticity in people with central nervous system diseases.

**Reference**	**Participants**	**Type of** **diseases**	**ESWT Protocol and other** **intervention**	**Treatment area**	**Clinical examination of** **spasticity**	**Outcome**
94	20 chronic poststroke patients	Stroke	– rESWT (1,500 shocks, 1.5 bar at 4 Hz, 1 session lasted about 6 min)	muscle belly of the wrist flexor hypertonic muscles of the forearm, the flexor carpi radialis, flexor carpi ulnaris.	MAS	A single session of rESWT could be an effective alternative treatment for reducing limb spasticity.
93	12 patients	Stroke	– rESWT (2,000 shocks, 1.0 bar at 5 Hz, 1 session)	gastrocnemius (plantar flexor spasm)	MAS, ankle PROM and AROM, PPFT, TUG	The rESWT improved plantar flexor spasticity, and the effects were sustained for 1 h.
90	rESWT group (n = 43) and the control group (n = 39)	CP	– rESWT (2,000 shocks, 2.0 bar at 10 Hz, probe diameter was 15 mm, 4 sessions, 1 week between sessions) – Both groups underwent routine rehabilitation (physiotherapy, occupational therapy, speech therapy, and orthotic treatment)	leg tricep hamstring and hamstring muscle belly with a radius of 2.5 cm	MAS, GMFM (88 items)	The rESWT combined with rehabilitation can quickly and effectively relieve paralysis of lower extremities, reduce the tension of hamstrings and calf muscles, relieve muscle spasms, and rapidly improve limb function.
88	25 children	CP	– rESWT (1,500 shocks, 1.5 bar at 5 Hz, 1 session) – Before the rESWT, a placebo session was applied, and each child served as its control	gastrocnemius and soleus muscle of the lower limb, mainly in the middle of the muscle belly	PROM of the ankle joint in degrees, MAS	A significant reduction in the spasticity of plantar flexor muscles after a single session of rESWT and this improvement remains at the 4-week follow-up.
95	rESWT group(*n =* 34) and placebo group(*n =* 34)	MS	– rESWT (2,000 shocks, 1.5 bar at 4 Hz, 4 sessions, 1 week between sessions) – placebo treatment – Both groups underwent drugs (baclofen, benzodiazepines, pregabalin, gabapentin, fampridine)	ankle extensors muscles	VAS, MAS, 10-MWT	Muscle tone was decreased 1 week after the last session and pain was decreased at all follow-up evaluations in rESWT group.
96	16 patients	MS	– rESWT (500 shocks, 1.8 bar at 4 Hz, 4 sessions, 1 week between sessions) – BoNT-A Injection (4 months after BoNT-A injection, the same spastic muscles were subjected to rESWT)	triceps surae	MAS, MTS, and kinematic analysis of passive and active ankle ROM	The use of rESWT following BoNT-A injection helps avoid some limitations and prolongs the therapeutic effects of BoNT-A therapy (a significant reduction of spasticity and improvement in passive and active ankle ROM).

rESWT, radial extracorporeal shock wave therapy; MAS, Modified Ashworth Scale; PROM, Passive range of motion; AROM, Active range of motion; CP, cerebral palsy; GMFM, Gross Motor Function Measure; MS, multiple sclerosis; VAS, Visual Analog Scale; 10-MWT, 10-meter walking test; MTS, Modified Tardieu Scale; PPFT, passive plantar flexor torque; TUG, timed up and go test. BoNT-A, Botulinum toxin serotype A.

## Cellular and molecular mechanism

**Figure 3:**
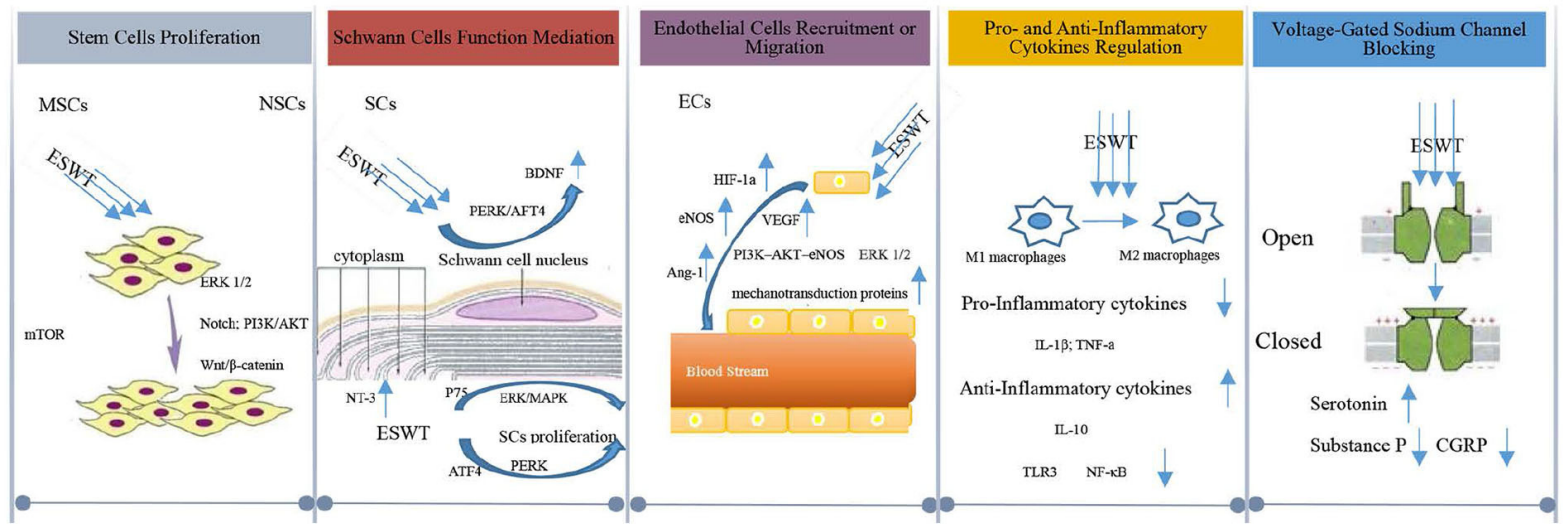
Cellular and molecular mechanisms for the interaction of ESWT with tissue.

At first, it was suggested that ESWT was likely to first cause microtrauma in nerve tissues and then accelerate the regeneration of neural tissues (97). Later, studies have shown that ESWT promoted neural tissue regeneration and repairment. It is generally believed that the cellular and molecular alterations triggered by ESWT can enhance the regeneration of injured tissues in the central and peripheral nervous systems. Next, we discuss the mechanism proposed to explain how ESWT triggers tissue alternation at the cellular and molecular level after the tissues are subjected to ESWT, and its effects on neural tissues (Figure 3).

### Stem cells proliferation

Although the ESWT effect on stem cells has gained increasing attention, the cellular and molecular mechanism of the stem cell growth triggered by ESWT remained poorly understood (98). It was found that ESWT could activate marrow stromal cells (MSCs) *in vitro* (99–101), causing accelerated proliferation to accomplish angiogenesis and nerve regeneration (102–104). In terms of the shock wave's function on MSCs proliferation, it was found that shock wave-elicited mechanical force triggered the mTORC1-FAK signaling pathway and remodeled the focal adhesion (FA) complex, inducing the MSC proliferation (105). MSCs comprise a heterogeneous group of stem/progenitor cells that can differentiate into mesoderm and non-mesoderm lineages, including epithelial cells and neurons, etc. (106). Moreover, MSCs have the multimodal therapeutic capacity in neural protection as well as the ability to promote angiogenesis in spinal cord injury (107). In the peripheral nerve system, MSCs might promote peripheral nerve regeneration after nerve injury (108).

When it comes to neural stem cells (NSCs), the ESWT's ability to promote the proliferation of NSCs was highly significant, which needs to be studied further. Kisoh et al. (109) suggested that recruitment of NSCs may improve brain dysfunction after cerebral ischemia. Zhang et al. (110) found that ESWT enhanced the proliferation and differentiation of NSCs by Notch, PI3K/AKT, and Wnt/β-catenin Signaling, leading to the repairment of the damaged nerve function in central nervous system diseases. It is known that NSCs play a crucial role in the central and peripheral nervous system, and could replace damaged neural cells (111–114). It is clear that stem cell proliferation induced by ESWT is of great significance in the repair and regeneration of the nervous system.

### Schwann cells function mediation

Schwann cells (SCs) exert a crucial function in the nervous system and are also involved in nerve regeneration and repairment (115, 116). It is believed that ESWT could first induce ATP release, stimulate purinergic receptors and activate ERK1/2 signaling, and then enhance SCs proliferation (117, 118), ultimately promoting nerve regeneration. Another study also suggested that low-intensity ESWT played a role in the proliferation of SCs *via* the activation of ERK/MAPK signaling and p75 neurotrophin receptor, enabling nerve regrowth (119). The proliferation of SCs triggered by ESWT leads to the upregulation of neurotrophic factors that are believed to enhance the process of nerve regeneration and functional recovery. Among neurotrophic factors, brain-derived neurotrophic factor (BDNF) has been shown to upregulate the function of several neurons in the nervous system, which could promote nerve regeneration *via* SC-dependent Janus kinase (JAK)/signal transducer and activator of transcription (JAK/STAT) pathways (120, 121). It was found that the expression level of BDNF in SCs *in vitro* increased when low-intensity ESWT was applied to nerve injury because ESWT could activate the protein kinase RNA-like endoplasmic reticulum (ER) kinase (PERK) pathway and enhance activating transcription factor 4 (ATF4) (47, 122). SCs can also maintain the survival of the neurons through the increased production of neurotrophic factor-3 (NT-3) and other neurotrophic factors around the injured tissues (123), ensuring continuous stimulation for the growth of axons in SCs (123).

### Endothelial cells recruitment or migration

ESWT can also have a significant impact on the neural tissue repairment and regeneration process by increasing local blood circulation, as ESWT can mechanically lead to vascular endothelial shear stress, generating a frictional force on the surface of the vascular epithelium (124), which can potentially lead to the recruitment or migration of endothelial cells (ECs). Studies of human umbilical vein endothelial cells (HUVECs) treated with ESWT (125) suggested that vascular endothelial growth factor (VEGF) and endothelial nitric oxide synthase (eNOS) are two essential angiogenesis mediators for ESWT-induced ECs recruitment and migration. Endothelial cells that are devoid of oxygen can increase the level of hypoxia-inducible factor 1 alpha (HIF-1α), thereby stimulating the release of VEGF. When VEGF binds to endothelial cells' receptors, it enhances the recruitment and migration of endothelial cells (126–128). In the nervous system, studies suggested that the increased expression of VEGF elicited by ESWT can produce a neuroprotective effect to reduce secondary neural tissue damage (62). Moreover, eNOS produces nitric oxide (NO), which contributes to vessel remodeling and vasodilating in the nervous system.

**Figure 4:**
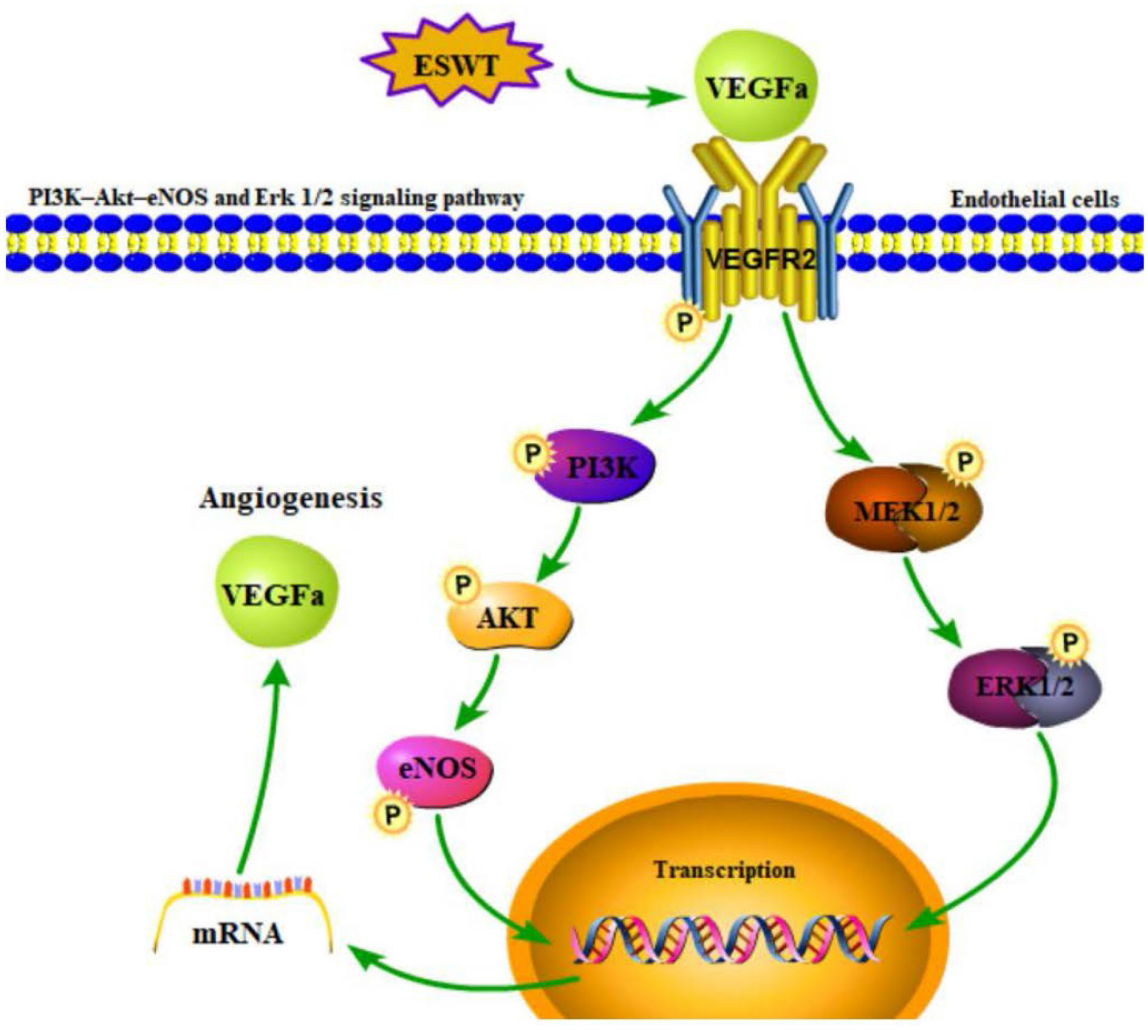
PI3K-AKT-eNOS and ERK 1/2 signaling pathway induced by ESWT could promote angiogenesis through endothelial cells migration.

It was suggested that the process of the recruitment or migration of ECs triggered by VEGF involved other proteins or signaling pathways. Hatanaka et al. (45) found that the shock wave treatment exhibited advantageous effects on angiogenesis by upregulating mRNA expression and protein levels of VEGF, eNOS, and mechanotransduction proteins including caveolin-1 and 1-integrin on its downstream pathways (ERK and AKT, et al.). The level of the downstream cytokines such as angiopoietin-1(Ang-1), a regulator of angiogenesis, increased with the level of eNOS due to ESWT. The increase of Ang-1 may also contribute to the recruitment and aggregation of ECs to the local site to form new blood vessels (129). Ha et al. (130) found that the PI3-AKT-eNOS and ERK 1/2 signaling pathways (Figure 4) were activated by shock waves, leading to ECs migration which enhances the process of angiogenesis. Consequently, when ESWT leads to blood supply recovery in ischemic neural tissues, neural tissues are regenerated and repaired.

### Pro- and anti-inflammatory cytokines regulation

ESWT could also mediate the expression of pro-and anti-inflammatory cytokines in a dose-dependent manner, reducing the pain and inflammation (131, 132). ESWT may exert the function of mediating inflammatory reactions through macrophages. Macrophages play a critical role in tissue regeneration and are shown to be sensitive to ESWT, which does not induce activation of resting macrophages, but reduces the induction of the pro-inflammatory profile in M1 macrophages and increases the anti-inflammatory profile with M2 macrophages (44, 133). Therefore, ESWT can reduce the concentration of pro-inflammatory cytokines such as TNF-α and IL-1β (134), while upregulating the expression of anti-inflammatory cytokines like IL-10 (135).

Macrophages also regulate immune and inflammatory responses by recognizing Toll-like receptors (TLRs), activating myeloid differentiation factor 88 (MyD88) and nuclear factor kappa-B (NF-κB) (136, 137). TLR3 signaling pathway perhaps modulates the early inflammatory response to ESWT in the nervous system and thus represents an innate mechanism of ischemic tissue regeneration and repairment (138–140). In the TLR3 signaling pathway, NF-κB is the downstream factor of TLR3. As a transcription factor, NF-κB represents a key mechanism for regulating the expression of multiple inflammatory genes. Hence, the suppression of NF-κB activation may account for the clinically beneficial action on tissue inflammation (141). It is believed that NF-κB activation in the nervous system could be inhibited by ESWT (142), which subsequently downregulates NF-κB and NF-κB-dependent inflammatory genes such as TNF-α and IL-1, thus leading to the modulation of the whole inflammatory process (132, 143–145). Regulating inflammatory reaction *via* pro-and anti-inflammatory cytokines balance triggered by ESWT results in a reduction in the inflammation of neural tissue injury and promotes neural repairment and neuroprotection.

### Voltage-gated sodium channel blocking

Voltage-gated sodium channels play a crucial role in pain transmission and sensitization pathways in the nervous system (146). ESWT is capable of closing the voltage-gated sodium channels to nociceptive input, which is competitively regulated at the spinal cord dorsal horn laminae (147). ESWT can activate serotonin in the cerebral cortex and reduce the production of substance P and calcitonin gene-related peptide (CGRP) in the dorsal root ganglion (25, 148, 149), which in turn leads to rapid degeneration of the intracutaneous nerve fibers to exert the effects of pain controlling (150). Substance P and CGRP are neuropeptides that are present in small-diameter afferent fibers. They are known to be involved in nociception in both the peripheral and central nervous systems (151). They can be released from nerve endings and play a pro-inflammatory role in the nerve tissues (152, 153). A reduction in their production by ESWT can ultimately prevent central sensitization and control the excessive neurons' excitement (154), thus resulting in pain-controlling effects beneficial to the sensory nerve regeneration processes (155).

## Conclusion and future outlook

Clinical areas, in which ESWT can be used, have expanded significantly, ranging from musculoskeletal diseases to regenerative medicine. Because ESWT is non-invasive, it can improve the conditions of many neurological disorders as well. In this review, we have discussed the essential characteristics of ESWT as well as the cellular and molecular mechanism proposed to explain how ESWT improves neural tissue repairment and regeneration in the nervous system. In addition, we reviewed both animal and clinical studies to show that ESWT can produce good therapeutic results when used to treat neurological disorders. For example, the animal studies reviewed show that treating cerebral infarction and spinal cord injury by ESWT could be safe if the optimal ESWT energy has been determined and used. The clinical studies reviewed show that ESWT produces good clinical results when used to treat CTS, TN, and PHN. Moreover, clinical studies have also shown that spasticity after central nervous system diseases such as stroke, cerebral palsy, and multiple sclerosis can be relieved as well by ESWT.

It should be noted that the use of ESWT to treat neurological disorders is promising and its potential is enormous, but there are many issues that are still needed to be addressed to better use ESWT clinically. For example, ESWT is not considered safe when used to treat bleeding disorders and pregnancy problems. In addition, pain, blistering, and hematomas can occur during and after treatment by ESWT although they may disappear within several days. Hence, any possible adverse reactions must be examined and suitable procedures to minimize or even eliminate these adverse effects must be developed before treatment of human diseases.

Another issue is a lack of a uniform standard for selecting the lesion area targeted by ESWT. As a result, the effectiveness of ESWT may vary among physicians. Moreover, when the lesion is deep, imaging such as ultrasound, X-ray, or MRI must be used to assist in locating the targeted area by ESWT. For example, when ESWT is used in the treatment of unresponsive wakefulness syndrome, the brainstem is targeted. Hence, it is necessary to combine body surface anatomical positioning and MRI to locate the targeted site on the body surface.

Moreover, as discussed above, high-energy ESWT can damage neural tissues. Moreover, the effectiveness of ESWT also depends on the penetration depth and the probe of the handpiece used in an ESWT device dictates the penetration depth. There are several ESWT device markers, each of which provides many different types of handpieces, making it difficult for physicians to decide and/or select which handpieces should be used to treat a given disease.

We believe that the biggest issue associated with the use of ESWT is a lack of a medical guideline for using ESWT for the treatment of different neurological disorders. Currently, physicians at different hospitals use ESWT for the treatment of neurological disorders based on their own experiences because of the lack of such a guideline. As a result, the effectiveness of ESWT may vary significantly among hospitals. Therefore, there is an urgent need for medical associations to develop medical guidelines to guide physicians in using ESWT to treat neurological disorders.

When it comes to the future basic research direction, a better understanding of the physical, cellular, and molecular mechanisms of interactions between ESWT and lesion tissues is urgently needed. For example, it has been suggested that ESWT may be able to transfer the mechanical signals to biological signals, thus triggering a series of events at the cellular and molecular levels, but it is still a mystery about how to complete this transition. A better understanding of such mechanisms will help physicians better use ESWT. In fact, the currently proposed cellular and molecular mechanism of the tissue's response to ESWT has already provided new insights into the use of ESWT to treat neurological disorders. It is our strong belief that with a better understanding of the interaction of ESWT with neural tissues in the nervous system, ESWT could, in the future, become a popular treatment option for many patients with central and peripheral nervous system diseases.

## Author contributions

JG and YM conceived and wrote the article. HH and YM proofread the data and provided guidance for writing and submission. All authors contributed to the article and approved the submitted version.

## Funding

This work was supported by the National Natural Science Foundation of China (81871840).

## Conflict of interest

The authors declare that the research was conducted in the absence of any commercial or financial relationships that could be construed as a potential conflict of interest.

## Publisher's note

All claims expressed in this article are solely those of the authors and do not necessarily represent those of their affiliated organizations, or those of the publisher, the editors and the reviewers. Any product that may be evaluated in this article, or claim that may be made by its manufacturer, is not guaranteed or endorsed by the publisher.
